# Extra-capsular growth of lymph node metastasis correlates with poor prognosis and high SOX9 expression in gastric cancer

**DOI:** 10.1186/s12885-018-4413-7

**Published:** 2018-04-27

**Authors:** Helena Link, Martin Angele, Miriam Schüller, Petra Ganschow, Lena Machetanz, Markus Guba, Jens Werner, Thomas Kirchner, Jens Neumann

**Affiliations:** 1 Institute of Pathology, Faculty of Medicine, LMU Munich, Thalkirchner Straße 36, 80337, Munich, Germany; 2 Department of General, Visceral, Transplantation, Vascular and Thoracic Surgery, Medical Center of the University of Munich, Munich, Germany; 3 German Cancer Consortium (DKTK), 69120 Heidelberg, Germany; 4 German Cancer Research Center (DKFZ), 69120 Heidelberg, Germany

**Keywords:** Gastric cancer, Lymph node metastasis, ECG, SOX9, Prognosis, Extranodal extension

## Abstract

**Background:**

Extra-capsular growth (ECG) describes the extension of neoplastic cells beyond the lymph node capsule. Aim of this study was to investigate the prognostic value of ECG and its association with a stem cell like phenotype indicated by expression of the transcription factor SOX9 in gastric cancer.

**Methods:**

By histological evaluation, 199 patients with nodal positive gastric cancer or adeoncarcinoma of the esophageal-gastric junction (AEG) were divided into two groups according to the presence (ECG) or absence (ICG) of extracapsular growth in at least one nodal metastasis. Of these, 194 patients were stained for SOX9 and SOX2 using immunohistochemistry. Seventeen nodal negative patients (pT3/4, pN0, pM0) served as controls.

**Results:**

Seventy-three patients (36.7%) showed ECG. ECG was associated with lower overall survival (*p* < 0.0001), advanced pT- (*p* = 0.03) and pN- category (p < 0.0001) and lymphovascular invasion (*p* = 0.014). In multivariate analysis, ECG was found to be an independent prognostic factor (HR = 2.1; 95% CI 1.7–3.4; *p* = 0.001). SOX9 expression correlated significantly with ECG (96% SOX9 high in ECG patients vs. 79% SOX9 high in patients with ICG; *p* = 0.002). Controls showed significantly reduced SOX9 expression compared to nodal positive carcinomas (59% vs. 85% high SOX9 expression; *p* = 0.006). No significant correlation of ECG and SOX2 (59% SOX2 negative in ECG patients vs. 64% in patients with ICG, *p* = 0.48) could be obtained.

**Conclusions:**

Patients with ECG exhibit poorer prognosis and ECG was found to be an independent prognostic factor. Thus, ECG turns out to be a morphological biomarker for a more aggressive phenotype in gastric cancer. This is supported by the fact that ECG correlates with the expression of SOX9, which has been described in the context of pro-oncogenic properties of tumours. However, the fact that SOX2 failed to show significant results indicate that ECG is not associated with a distinct cancer stem cell phenotype in gastric cancer.

## Background

The extra-capsular growth (ECG) describes the extension of neoplastic cells beyond the lymph node capsule into the perinodular soft tissue. Being a prognostic factor for cancer in several organs [1, 2] including the gastrointestinal tract [3, 4], ECG has already found access to staging systems such as the squamous cell carcinoma of the vulva and the head and neck [5].

A systematic review about colorectal cancer determined ECG to be associated with poorer prognosis and a higher risk of recurrence of disease [6]. Therefore, the parameter ECG is also suggested to be included as a prognostic parameter in colorectal cancer staging systems [6]. In gastric cancer, ECG is associated with poorer 5-year survival and was also found to be an independent prognostic factor [4, 7]. ECG is associated with larger tumour size, advanced pT- and pN-category and lymphovascular and perineural invasion [4, 7].

Both SOX9 and SOX2 are members of the DNA- binding HMG domain proteins and part of the SOX-family of transcription factors. SOX2 is mainly involved in the regulation of embryonic development and in the determination of cell fate. Besides being required for the stem-cell maintenance in the central nervous system, SOX2 is known to regulate gene expression in the stomach [8]. In gastric cancer, high expression of SOX2 was found to be associated with decreased rates of lymph node metastasis and better treatment outcome [9]. SOX9 is important for sex determination as it initiates testis development. It also plays a decisive role in chondrogenesis by regulating cartilage specific gene expression [10, 11]. In the gastrointestinal tract, SOX9 is expressed in nuclei of crypt cells including Paneth and stem cells [11], playing an important role for endoderm development and homeostasis of the intestinal epithelium by interacting with the Wnt/ß-catenin-signalling pathway. SOX9, together with the Wnt/ß-catenin-target Slug, was found to determine the stem cell state in mammary cells by inducing differentiated luminal cells to pass through epithelial-mesenchymal transition (EMT), thus driving malignant progression an metastasis [12].

SOX9 is associated with poorer prognosis and advanced TNM category in different types of gastrointestinal cancer [13, 14]. In colorectal cancer, high SOX9 expression was found to be an independent prognostic factor [14]. Similar, in gastric cancer SOX9 over-expression is related to tumour progression and associated with lower 5-year survival, tumour invasion as well as advanced TNM category [15].

Based on these findings it was the aim of our study to analyse the prognostic value of ECG in gastric cancer as a morphological marker for a more aggressive tumour phenotype. Furthermore, we intended to analyse the association of ECG with the protein expression of the embryonic transcription factors SOX9 and SOX2 both being potential biomarkers for a stem cell like phenotype in gastric cancer.

## Methods

### Tissue selection

From the data bank of the Department of General, Visceral, Transplantation, Vascular and Thoracic Surgery, Medical Center of the University of Munich, 529 patients who underwent surgery for gastric cancer or adenocarcinoma of the esophageal-gastric junction (AEG) between 2002 and 2014 were selected. Cases with lymph node metastasis were extracted (*n* = 260) and the histological slides of 199 of the 260 nodal positive cases could be collected from the archives of the Institute of Pathology, Faculty of Medicine, LMU Munich. Clinico-pathological parameters are summarized in Table 1. The lymph node status was evaluated according to standard protocols following the national German guidelines. In brief, all lymph nodes included in the surgical specimen were dissected. Lymph nodes larger than 0.5 cm were divided in half and from each lymph node specimen two to three serial sections were prepared. In indistinct cases additional serial sections were made.

**Table 1:** Correlation of the ECG/ICG-status with clinico-pathological variables at time of surgery (according to TNM Classification of Malignant Tumours 8th edition 2017). Percent-values are given in parentheses

		Capsule status		
Characteristic	Total (%)	ECG	ICG	p
All patients	199 (100)	73 (36.7)	126 (63.3)	
Age (median. 66.0) years				
≤ 66	100 (50.3)	33 (16.6)	67 (33.7)	0.279
≥ 67	99 (49.7)	40 (20.1)	59 (29.6)	
Gender				
Male	128 (64.3)	41 (20.6)	87 (43.7)	0.067
Female	71 (35.7)	32 (16.1)	39 (19.6)	
Lauren classification				
diffuse	69 (34.7)	33 (16.6)	36 (18.1)	0.059
intestinal	108 (54.3)	33 (16.6)	75 (37.7)	
mixed type	22 (11.1)	7 (3.5)	15 (7.5)	
Tumor size (UICC)				
T0	4 (2.0)	1 (0.5)	3 (1.5)	**0.032**
T1	2 (1.0)	0 (0)	2 (1.0)	
T2	97 (48.7)	27 (13.6)	70 (35.2)	
T3	61 (30.7)	25 (12.6)	36 (18.1)	
T4	34 (17.1)	19 (9.5)	15 (7.5)	
Tx	1 (0.5)	1 (0.5)	0 (0)	
Nodal status				
N0	0 (0)	0 (0)	0 (0)	**–**
N+	199 (100)	73 (36.7)	126 (63.3)	
Distant metastasis				
M0	126 (63.3)	41 (20.6)	85 (42.7)	0.179
M1	58 (29.1)	27 (13.6)	31 (15.6)	
Mx	15 (7.5)	5 (2.5)	10 (5.0)	
Tumor grade (WHO)				
G1	2 (1.0)	1 (0.5)	1 (0.5)	0.207
G2	31 (15.6)	7 (3.5)	24 (12.1)	
G3	162 (81.4)	63 (31.7)	99 (49.7)	
Gx	4 (2.0)	2 (1.0)	2 (1.0)	

Significant results are indicated by bold numbers

**Fig. 1:**
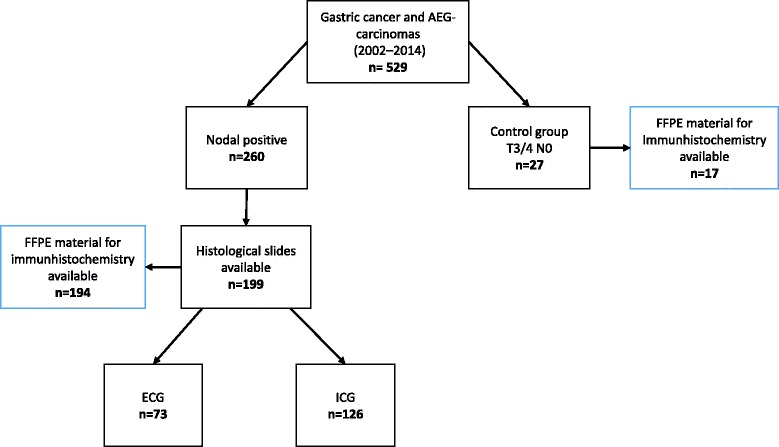
Flowchart showing the selection of nodal positive and nodal negative cases for histological evaluation of extra-capsular growth (ECG) and intra-capsular growth (ICG) as well as for immunohistochemical analysis of SOX9 expression

**Fig. 2:**
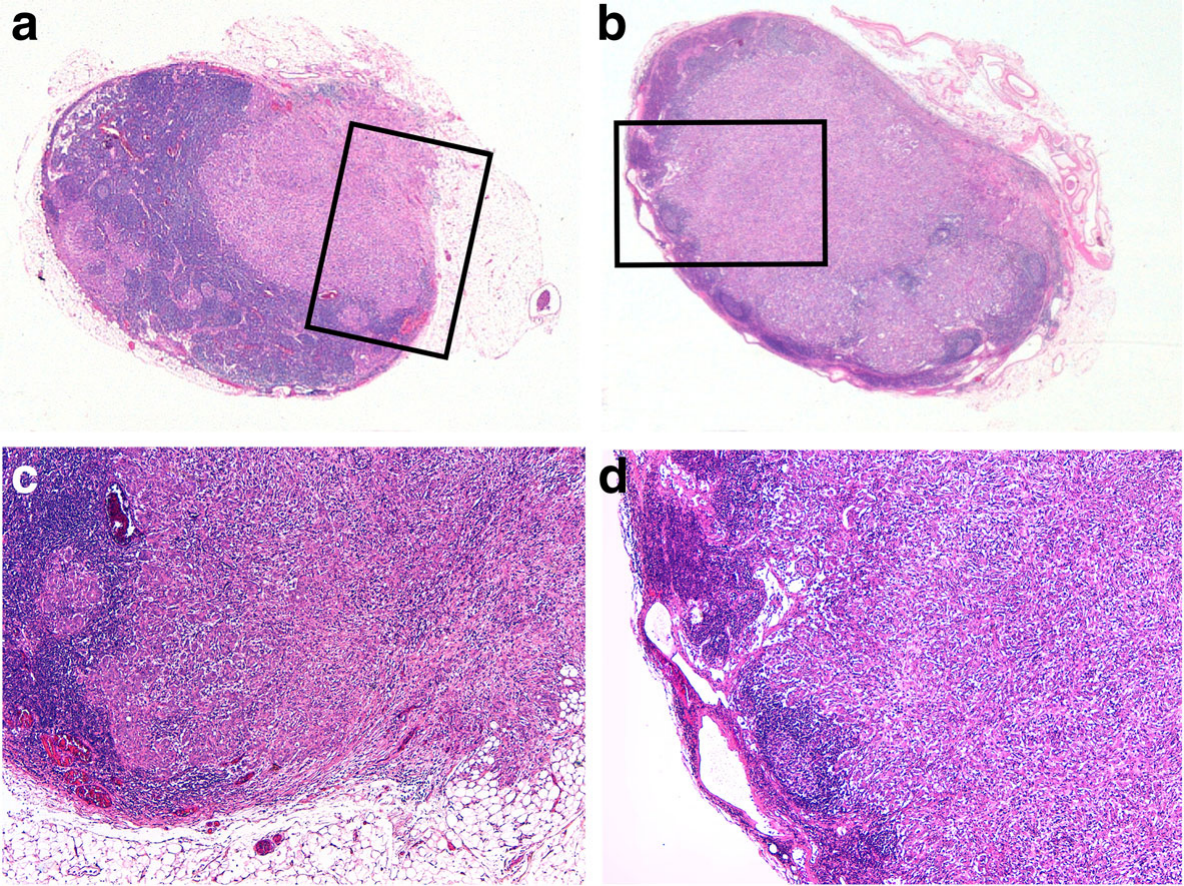
Overview of lymph-node metastasis of gastric cancer with ECG (**a**) and ICG (**b**). The frames indicate areas shown with higher magnification in **c** and **d** (200 fold, hematoxylin and eosin stain), respectively

Formalin fixed and paraffin embedded (FFPE) specimens of the primary tumours of 194 of the 199 nodal positive patients were collected for immunohistochemistry. Seventeen locally advanced, nodal negative patients without distant metastasis (pT3/4, pN0, pM0) served as controls (Fig. 1). The histological slides of all lymph node positive cases (*n* = 199) were evaluated by two independent observers (JN and HL) according to the presence (ECG) or absence (ICG) of extracapsular growth in at least one nodal metastasis (Fig. 2a-d).

### Immunohistochemistry

Immunohistochemical stainings were performed using 5 μm serial tissue sections of representative FFPE tumour samples. For SOX9 specific immunohistochemistry first a heat-induced epitope retrieval was done applying ProTaqs IV Anitgen-Enhancer (Quartett, Germany) before adding primary SOX9 specific rabbit monoclonal antibody (1:100, SOX9 clone D8G8H, Cell Signalling Technology, Danvers, MA) for 60 min at room temperature. Thereafter, a development step was introduced by adding detection-system (Vectastain ABC-Kit Elite Universal, Biozol, Germany) and subsequently substrate-chromogen containing DAB+ system (DAKO, Germany) according to the respective manufacturer’s protocols.

For SOX2 specific immunohistochemistry heat-induced epitope retrieval was performed applying Epitope Retrieval Solution (Novocastra, Germany) followed by adding primary SOX2 specific rabbit monoclonal antibody (1:50, SOX2 clone D6D9, Cell Signalling Technology, Danvers, MA) for 60 min at room temperature. The development step was made by adding the detection-system (ImmPRESS Reagent Kit Anti-RABBIT Ig, Vector, Germany) and substrate-chromogen containing DAB+ system (DAKO, Germany).

Finally, for both markers slides were counterstained using Hematoxylin (Vector Laboratories, Burlingame, CA). To control for unspecific binding of antibodies, isotype controls were included (data not shown).

### Scoring of immunohistochemistry

**Fig. 3:**
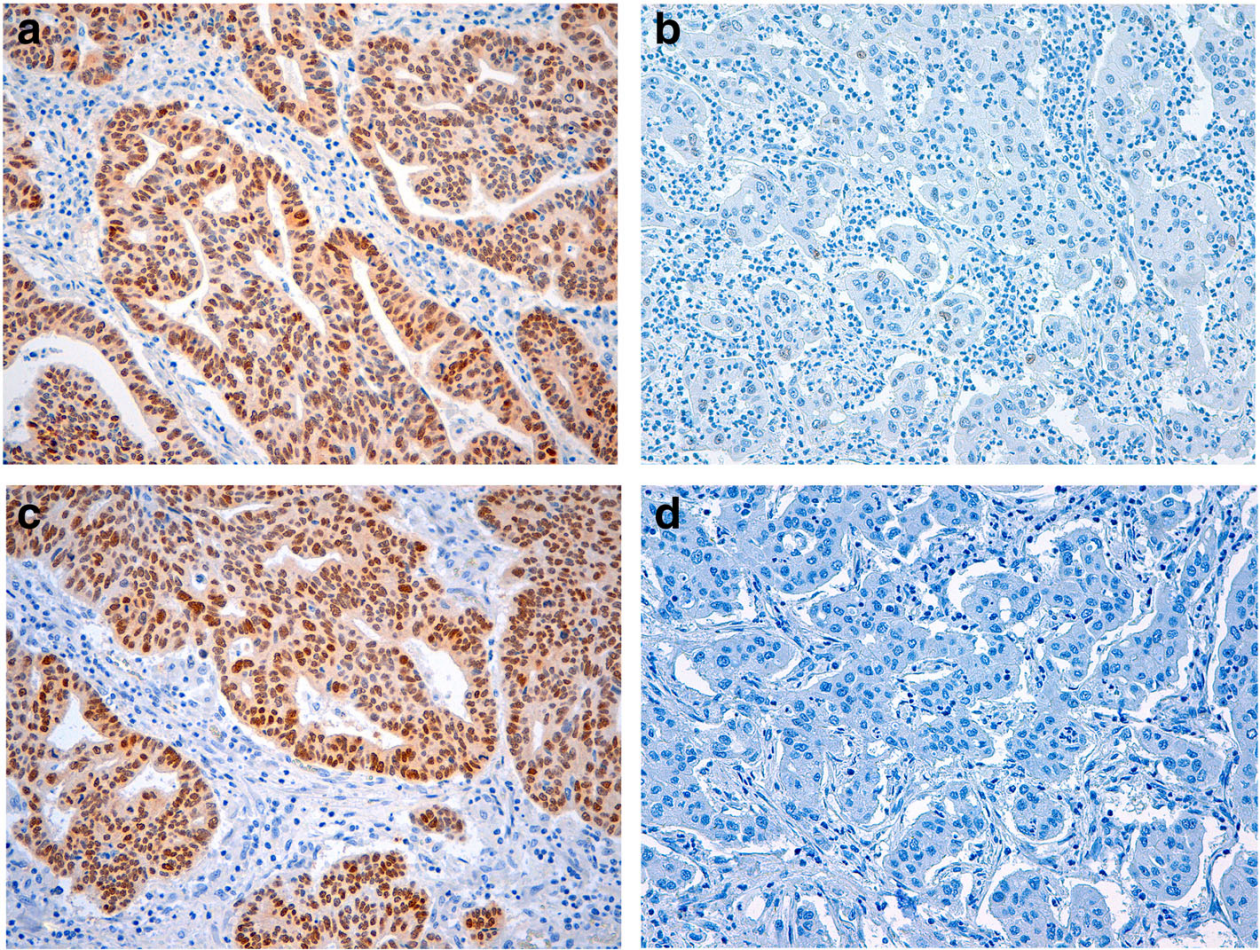
Immunhistochemical staining of SOX9 and SOX2 in gastric adenocarcinoma (200-fold magnification). In **a** adenocarcinoma with high and in **b** with low immunohistochemical expression of SOX9 are shown. Adenocarcinoma with positive and negative SOX2 expression is shown in **c** and in **d**, respectively

Immunohistochemistry both for SOX9 and SOX2 were evaluated independently by two observers (JN and HL). Nuclear staining was reported for both, percentage and intensity of stained nuclei throughout the tumour. Intensity values were categorized as follows: no staining – 0, weak–1, moderate – 2 and strong – 3. The staining of SOX9 was evaluated as follows: when less than 30% of tumour cells were stained and/or the intensity was missing (0) or weak (1), immunostaining was reported to be negative (Fig. 3a and b). When less than 10% of tumour cells were stained for SOX2 and/or the intensity was missing (0) or weak (1), immunostaining was reported to be negative (Fig. 3c and d). Discrepant cases were discussed and a consensus was reached.

### Statistics

All statistics were calculated using SPSS v.17.0 (SPSS Inc.). Significance of correlations of the immunhistochemical analyses was calculated using the χ^2^-test. Survival analysis was performed using Kaplan-Meier-test and multivariate Cox-regression. For all tests a *p*-value of *p* < 0.05 was considered statistically significant.

## Results

**Fig. 4:**
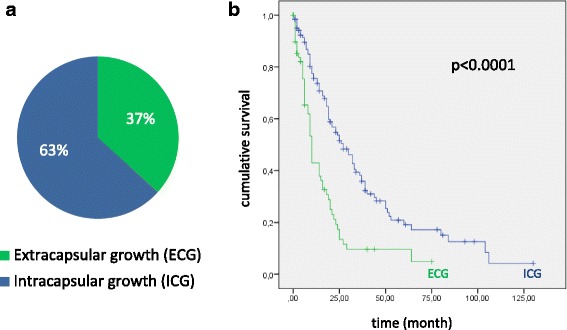
Proportion of cases with ECG and ICG (**a**) and its correlations with overall survival (**b**)

The average number of the lymph nodes being observed was 24.4 (min. 3, max. 85). Seventy-three patients (36.7%) showed ECG (Fig. 4a). The mean number of lymph nodes with ECG was 2.6 (min. 1, max. 12). Presence of ECG was associated with significantly lower overall survival (*p* < 0.0001; Fig. 4b). Patients with ECG had a median survival of 16.21 months (95% confidence interval (CI) 11.69–20.74; standard deviation (SD) 2.31) whereas patients without ECG had a median survival of 37.07 months (95% CI 30.28–43.85;SD 3.46). In univariate analysis, cox-regression detected a hazard ratio (HR) of 2.4 (95% CI 1.7–3.4; *p* < 0.0001).

Regarding the subgroup of stage UICC II patients (*n* = 76), ECG was also associated with significantly lower overall survival (*p* = 0.001). The median survival of stage II patients with ECG (*n* = 16) was 21.0 months (95% CI 11.3–30.6; SD 5.0) whereas stage II patients with ICG (*n* = 60) had a median survival of 50.7 months (95% CI 39.0–62.4; SD 6.0). In univariate cox-regression, the HR was 2.9 (95% CI 1.5–5.6; *p* = 0.001). In multivariate analysis, ECG was found to be a prognostic factor (HR = 2.1; 95% CI 1.7–3.4; *p* = 0.001), independent from T- and N-category, UICC-stage and lymphangiosis carcinomatosa.

**Fig. 5:**
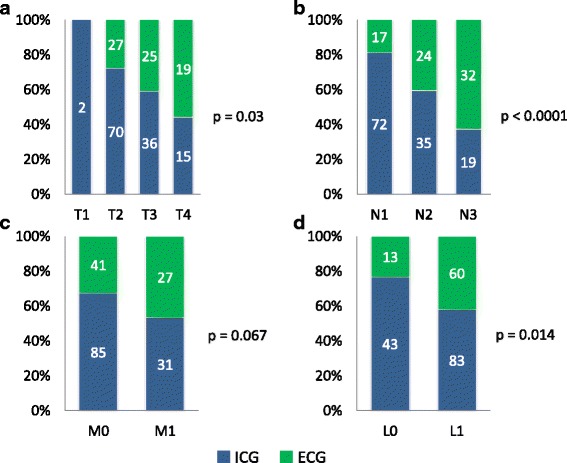
Correlation of ECG with pT- (**a**) and pN-category (**b**), presence (pM1) or absence (pM0) of distant metastases (**c**) and with presence (L1) or absence (L0) of lymphangiosis carcinomatosa (**d**) according to TNM-classification of malignant s (8th edition 2017)

Presence of ECG was associated with advanced pT- (*p* = 0.03; Fig. 5a) and pN-category (*p* < 0.0001; Fig. 5b) but not with the presence of distant metastases (pM category, *p* = 0.067; Fig. 5c). In category pT2 (*n* = 97), 28% showed an ECG, whereas in category pT3 (*n* = 61) it was 41% and in pT3 (*n* = 34) even 56% (p = 0.03). The association with pN-category was highly significant (*p* < 0.0001); the percentage of cases with ECG doubled from category pN1 to pN2 and tripled from pN1 to pN3 (pN1: *n* = 89, ECG 19%; pN2: *n* = 59, ECG = 41%; pN3: *n* = 51, ECG = 63%).

ECG was further associated with lymphovascular invasion. Forty-two percent of cases with lymphangiosis carcinomatosa (L1, *n* = 143) showed ECG whereas among patients without lymphangiosis carcinomatosa (L0, *n* = 56) it was only 23% (Fig. 5d). The majority of nodal positive cases were classified as SOX9-high positive (*n* = 165, 85%; Fig. 3a). Twenty-nine patients showed low or missing SOX9 expression and were classified as negative (*n* = 29, 15%; Fig. 3b). Ten of seventeen (59%) control cases showed high positive SOX9 expression. In contrast to these results, the majority of nodal positive cases were classified as SOX2-negative (*n* = 120, 62%, Fig. 3d). 74 patients showed positive SOX2 expression (*n* = 74, 38%, Fig. 3c). Ten of seventeen (59%) control cases showed negative SOX2 expression.

**Fig. 6:**
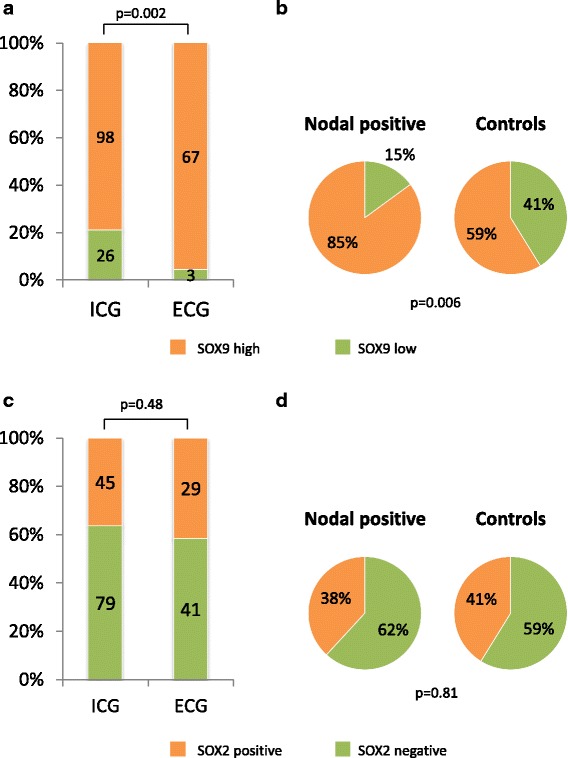
Correlation of SOX9 and SOX2 expression with ECG and ICG (**a, c**) and with the presence or absence of lymph-node metastasis (**b, d**)

SOX9 expression correlated significantly with ECG (96% high positive SOX9 expression in ECG patients vs. 79% high positive SOX9 expression in ICG patients; *p* = 0.002; Fig. 6a). The controls showed a significantly lower rate of high positive SOX9 expression compared to the nodal positive carcinomas (nodal positive: 85% high positive SOX9 expression; control: 59% (10/17); *p* = 0.006; Fig. 6b). Correlations of SOX9 expression with clinic-pathological parameters are shown in Table 2. There was no significant correlation between SOX2 expression and ECG (59% negative SOX2 expression in ECG patients vs. 64% negative SOX2 expression in ICG patients, *p* = 0.48, Fig. 6c). Neither SOX9 nor SOX2 expression were associated with poorer prognosis (data not shown).

**Table 2:** Correlation of SOX9 expression with clinico-pathological parameters at time of surgery (according to TNM Classification of Malignant Tumours 8th edition 2017). Percent-values are given in parentheses

		SOX9		
Characteristic	Total (%)	high positive	negative	p
All patients	211 (100)	175 (82.9)	36 (17.1)	
Age (median. 66.5) years				
≤ 66	104 (49.3)	81 (38.4)	23 (10.9)	0.054
≥ 67	107 (50.7)	94 (44.5)	13 (6.2)	
Gender				
Male	135 (64.0)	111 (52.6)	24 (11.4)	0.712
Female	76 (36.0)	64 (30.3)	12 (5.7)	
Lauren classification				
diffuse	74 (35.1)	58 (27.5)	16 (7.6)	0.290
intestinal	113 (53.6)	98 (46.4)	15 (7.1)	
mixed type	24 (11.4)	19 (9.0)	5 (2.4)	
Tumor size (UICC)				
T0	4 (1.9)	2 (0.9)	2 (0.9)	0.286
T1	2 (0.9)	2 (0.9)	0 (0)	
T2	96 (45.5)	81 (38.4)	15 (7.1)	
T3	73 (34.6)	58 (27.5)	15 (7.1)	
T4	35 (16.6)	31 (14.7)	4 (1.9)	
Tx	1 (0.5)	1 (0.5)	0 (0)	
Nodal status				
N0	17 (8.1)	10 (4.7)	7 (3.3)	**0.006**
N+	194 (91.9)	165 (78.2)	29 (13.7)	
Capsule status of N+ cases				
extra-capsular growth (ECG)	70 (33.2)	67 (31.8)	3 (1.4)	**0.0003**
intra-capsular growth (ICG)	124 (58.8)	98 (46.4)	26 (12.3)	
Distant metastasis				
M0	137 (64.9)	111 (52.6)	26 (12.3)	0.565
M1	58 (27.5)	49 (23.2)	9 (4.3)	
Mx	16 (7.6)	15 (7.1)	1 (0.5)	
Tumor grade (WHO)				
G1	2 (0.9)	2 (0.9)	0 (0)	0.816
G2	31 (14.7)	26 (12.3)	5 (2.4)	
G3	173 (82.0)	144 (68.2)	29 (13.7)	
Gx	5 (2.4)	3 (1.4)	2 (0.9)	

Significant results are indicated by bold numbers

## Discussion

Extracapsular growth has become an important part of oncological studies in the last years. The association of ECG and poorer prognosis, both in gastric cancer [4] and in gastrointestinal malignancies in general [3], turns the parameter ECG into an interesting target for recent studies regarding tumours of the gastrointestinal tract [5, 16, 17]. Its association with histomorphological parameters such as TNM-category, lymphovascular invasion and tumour grading [6, 7] implies ECG to be a morphological marker for a more aggressive biological phenotype of gastrointestinal cancer. Recently, extranodal extension already became a part of the lymph node category of head and neck cancer staging systems [18, 19]. Therefore, there is huge evidence that ECG should find access to tumour staging classifications of gastrointestinal malignancies in addition to the established staging systems TNM and UICC.

Our results are in concordance with previously published data. In this study, we could confirm ECG to be associated with poorer prognosis and multivariate analysis identified ECG to be an independent prognostic factor. In addition, the yet known correlation of ECG and histomorphological parameters could be retraced in this study as ECG was significantly associated with higher pT- and pN- categories and the presence of lymphovascular invasion. Thus, from the accordance of our results with recently published data it may be concluded that the results of this study are representative for gastric cancer patients in general.

Since ECG seems to be a morphological marker for a more aggressive tumour phenotype there may be an association of ECG with other markers for aggressive growth. Of interest, we could not obtain a correlation of ECG and Lauren classification, although diffuse-type carcinomas are known to grow aggressively and to be associated with bad prognosis [20]. Thus, it could be concluded that ECG is more likely associated with intrinsic factors such as pro-oncogenic properties of tumour cells. In this study, we analysed two different markers associated with stem cell features of tumour cells in the literature. As our data show a significant correlation between ECG and the marker SOX9, it could be assumed from our data that ECG may be associated with stemness properties in gastric cancer. SOX9 turned out to be an important stem cell marker for gastrointestinal and other malignancies. In mammary cell carcinoma, SOX9 is known to be partly responsible for the determination of the stem cell state by inducing epithelial mesenchymal transition (EMT) [12], which is also known to play a decisive role in the genesis of gastrointestinal tumours [21, 22]. Furthermore, high SOX9 expression is associated with advanced TNM category, poorer prognosis and tumour progression, both in gastric cancer and other types of gastrointestinal cancer [13–15, 23]. The deletion of SOX9 results in an increased epithelial proliferation and intestinal hyperplasia [10, 24]. Nevertheless, it remains controversial, whether SOX9 is a suitable stem cell marker in gastric cancer. In our collection of gastric cancer patients we could demonstrate a significant association of ECG and SOX9 but no correlation between ECG and SOX2, which is an established stem cell marker in gastric cancer [25]. Thus, we could not proof a correlation of EGC with a distinct stem cell phenotype. However, it could be concluded from our data that SOX9 acts as a pro-oncogene in gastric cancer and plays an important role in gastric cancer progression and formation of ECG in lymph node metastasis.

In this study, the expression of SOX9 was neither associated with survival nor with pT- or pM-category which is mainly due to the fact that our data primarily includes nodal positive patients and is therefore biased. However, our data did show a significant association of high SOX9 expression and nodal status and the presence of ECG. From the fact that cases with extracapsular growth show higher SOX9 expression it can be concluded that ECG is a promising biomarker for a more aggressive gastric cancer phenotype. Further studies, especially on molecular levels need to be done in order to understand the functional and biological aspects of ECG and the intrinsic significance and exact mechanism of pro-oncogenes such as SOX9.

## Conclusions

In summary, our data confirm that ECG is an independent prognostic factor and a potential morphological biomarker for a more aggressive phenotype in gastric cancer. This is supported by the fact that ECG correlates with the expression of SOX9 indicating that this biomarker probably plays an important role in the pathogenesis of gastric cancer and ECG formation. Thus, it could be concluded from our data that in gastric cancer ECG, similar to tumour of the vulva and the head and neck region, should be integrated in established cancer staging systems such as TNM and UICC.

## Abbreviations

AEG: Adenocarcinoma of the esophageal-gastric junction
CI: Confidence interval
ECG: Extra-capsular growth
EMT: Epithelial-mesenchymal transition
FFPE: Formalin fixed and paraffin embedded
HR: Hazard ratio
ICG: Intracapsular growth
SD: Standard deviation
